# Deletion of Nrf2 Enhances Susceptibility to Sinonasal Inflammation After Short-Term PM_2.5_ Exposure

**DOI:** 10.1002/alr.23550

**Published:** 2025-02-27

**Authors:** Anuj Tharakan, Asiana Gurung, Nyall R. London, Stefany Lazieh, Shyam Biswal, Murugappan Ramanathan

**Affiliations:** 1 Department of Otolaryngology-Head and Neck Surgery, Johns Hopkins University School of Medicine, Baltimore, Maryland, USA; 2 Department of Environmental Health and Engineering, Johns Hopkins Bloomberg School of Public Health, Baltimore, Maryland, USA

**Keywords:** air pollution, Nrf2, PM_2.5_, short-term exposure

## Abstract

**Background:**

Particulate matter 2.5 (PM_2.5_) has been identified as one of the most pathogenic components of air pollution and has been associated with chronic rhinosinusitis (CRS) prevalence and severity. Nuclear erythroid 2-related factor 2 (Nrf2), an antioxidant transcription factor, is critical for protective responses against environmental exposures, such as PM_2.5_. The goal of this study was to elucidate the role of Nrf2 in a short-term PM_2.5_-induced murine model of CRS.

**Methods:**

C57BL/6 wild-type (*n* = 12) and Nrf2-deficient (*n* = 12) mice were intranasally challenged with 400 μg of PM_2.5_ or saline alone for 2 weeks. Heads were harvested and sectioned to perform IHC and flow cytometry, and sinonasal mucosa was dissected to perform qPCR.

**Results:**

Nrf2^−/−^ mice exhibited a 50% increase in sinonasal inflammation, with a neutrophilic predominance. Nrf2 deficiency also promoted elevated levels of sinonasal *Muc5ac* expression and increased expression of type 2, type 3, and epithelial-derived cytokines.

**Conclusion:**

This study demonstrates that the Nrf2 antioxidant pathway is critical in controlling short-term PM_2.5_-induced sinonasal type 3 inflammation and may represent a potential therapeutic target to modulate the inflammatory response induced by air pollution. This is the first study to our knowledge to demonstrate that Nrf2 regulates PM_2.5_-induced rhinosinusitis in vivo.

## 1 | Introduction

Chronic rhinosinusitis (CRS) is an inflammatory disease of the nasal and paranasal sinuses that affects 10%–15% of the population [1]. CRS is a heterogenous disorder and can be classified based on its underlying immune mechanisms and the presence or absence of nasal polyps [2]. Generally, CRS with nasal polyposis (CRSwNP) is driven by type 2, eosinophil-predominant infiltrate, whereas CRS without nasal polyps (CRSsNP) is mediated by type 1 and type 3 immune mechanisms, promoting neutrophilic mucosal inflammation [2, 3]. The etiology of CRS is not well understood, but exposure to airborne particulates has been linked to CRS incidence and severity [4–6].

Airborne particulate matter (PM) consists of a mixture of solid and liquid particles and is typically categorized based on particle size [7]. Fine PM (PM_2.5_) is considered the most harmful type of particulate due to its ability to penetrate into tissues and cause cellular dysfunction [8, 9]. Previous studies have demonstrated that airway exposure to PM_2.5_ in animal models elicits a potent type 3 immune response in the sinonasal mucosa, similar to the inflammatory process seen in CRSsNP [10–12].

PM contains redox active compounds including polycyclic aromatic hydrocarbons, heavy metals, and nitrogen oxides, which promote tissue injury via the induction of reactive oxygen species (ROS) production [7, 13, 14]. The nuclear factor erythroid 2-related factor 2 (Nrf2) pathway is a key endogenous mechanism that mitigates cellular oxidative stress by regulating the expression of antioxidant genes [15]. These genes, such as heme oxidase (HMOX-1), glutamate cysteine ligase catalytic subunit (GCLC), and glutamate cysteine ligase modulatory subunit (GCLM), encode proteins that regulate glutathione recycling and iron homeostasis [15, 16].

One of the key drivers of CRS pathogenesis is sinonasal epithelial barrier function [17–20]. Previously, our group demonstrated that epithelial barrier dysfunction induced by PM_2.5_ and other environmental stimuli could be reversed via activation of the Nrf2 pathway [18, 21, 22]. Additional studies revealed that chronic PM_2.5_ exposure downregulated expression of Nrf2 target genes by inducing epigenetic changes [23]. Here, we utilize a murine subacute PM_2.5_ exposure model in Nrf2 wild-type and Nrf2-deficient mice to explore the role of Nrf2 on particulate-induced inflammation in vivo. We find that Nrf2 deficiency significantly exacerbates sinonasal and olfactory neutrophilic inflammation, increases type 2 and type 3 cytokines, and increases epithelial mucin gene synthesis.

## 2 | Methods

### 2.1 | Animals

All animal procedures were approved by the Johns Hopkins Institutional Animal Care and Use Committee (IACUC). Nrf2-deficient mice were purchased from Jackson Laboratory (Bar Harbor, ME) and were backcrossed to C57BL6/J Nrf2 wild-type mice for five generations or more. Male homozygous Nrf2^−/−^ mice and littermate Nrf2^+/+^ mice at least 6 weeks of age were used in this study. Animals were housed in a specific pathogen-free facility with single ventilation.

### 2.2 | PM_2.5_ Exposure

PM used for exposure was collected by a 3-stage cyclone system [24]. Only the PM from stage 3 was used for the exposure. Stage 3 of the cyclone system collects PM in the size range of 0.3–3.5 μm. The PM was collected on the Johns Hopkins School of Public Health campus in Baltimore, MD (i.e., an ambient urban environment) for 23 days during fall of 2012. Particles were resuspended at in phosphate-buffered saline (PBS) at a concentration of 400 μg/mL. Mice were exposed to 400 μg of PM_2.5_ daily for 14 days. On day 15, mice were sacrificed and processed for analysis.

### 2.3 | qPCR

Total RNA was isolated from mouse sinonasal tissue using Direct-zol RNA MiniPrep (Zymo Research, Irvine, CA, USA). Equal amounts of RNA were transcribed into cDNA by a high-capacity cDNA reverse transcription kit (Applied Biosystems ref 4368814, Waltham, MA, USA). Note that 1000 nanograms of cDNA were added to a 20-μl PCR reaction using TaqMan Fast Universal PCR Master Mix (Applied Biosystems, Waltham, MA, USA) and analyzed on a StepOne Plus System (Applied Biosystems). Fold change in mRNA expression was calculated using the comparative cycle method (2^−ΔΔCt^). TaqMan probes were purchased from Life Technologies Corp (Carlsbad, CA, USA) for the following targets: Il4, Il5, Il13, Il25, Il33, Il6, Il17, and Muc5ac. All qPCR experiments were performed in duplicate.

### 2.4 | Immunofluorescence

Twelve microliters of cryosections were washed in 1x PBS and then blocked in 10% normal serum containing 0.1% Triton X-100, followed by incubation with primary antibodies at 4°C overnight. After washing in PBS, the tissue sections were incubated with Alexa fluor-conjugated secondary antibodies along with DAPI for nuclear counterstaining. The following primary antibodies were used: chicken anti-keratin-5 (Krt5) (1:500, 905903; BioLegend, San Diego, CA, USA) and rat anti-CD45 (1:550, Invitrogen).

Fluorescent immunostaining images were obtained using a Zeiss LSM 780 confocal microscope. The average mean gray intensity of immunofluorescence was calculated in two Nrf2^+/+^ control and two Nrf2^−/−^ transgenic mice using the Zen lite 3.9 (Zeiss) software and plotted with standard error of the mean.

### 2.5 | Flow Cytometry

Mouse sinonasal tissues were isolated and single cell suspensions were generated as previously described [23]. Tissue samples were minced and incubated for 45 min in 6 mL of dissociation buffer consisting of RPMI 1640, penicillin/streptomycin, *β*-mercaptoethanol, 0.2 mg/mL DNase I (Sigma Aldrich), and 50 μg/mL Liberase TL (Roche Diagnostics, Indianapolis, IN). To make a single-cell suspension, the tissue was forced through a 70-μm cell strainer. Dissociation was stopped by the addition of 600 μL of fetal bovine serum. Cells were stained with fixable viability dye (Zombie Aqua, Biolegend, San Diego, CA) before immunostaining. Immunostaining was performed by incubating cells with Fc-block for 10 min, followed by incubation with anti-Siglec-F and anti-Ly6G antibodies for 15 min. Cells were then washed and analyzed on an LSRII flow cytometer (BD Biosciences). Eosinophils were identified as Siglec-F^+^ , Ly6G^−^ , and SSC^hi^ cells. Neutrophils were identified as Siglec-F^−^, Ly6G^+^, and SSC^lo^ cells.

### 2.6 | Statistical Analysis

All values are presented as mean ± SEM. Statistical significance was determined by Student’s *t*-test or Mann–Whitney *U* test by comparing PM-exposed Nrf2^+/+^ and Nrf2^−/−^ mice. Statistical significance was considered *p* < 0.05. Results were analyzed with the use of Prism 10 (GraphPad, La Jolla, CA).

## 3 | Results

### 3.1 | Nrf2 Deficiency Exacerbates Sinonasal Inflammation in PM Exposed Mice

**FIGURE 1 |:**
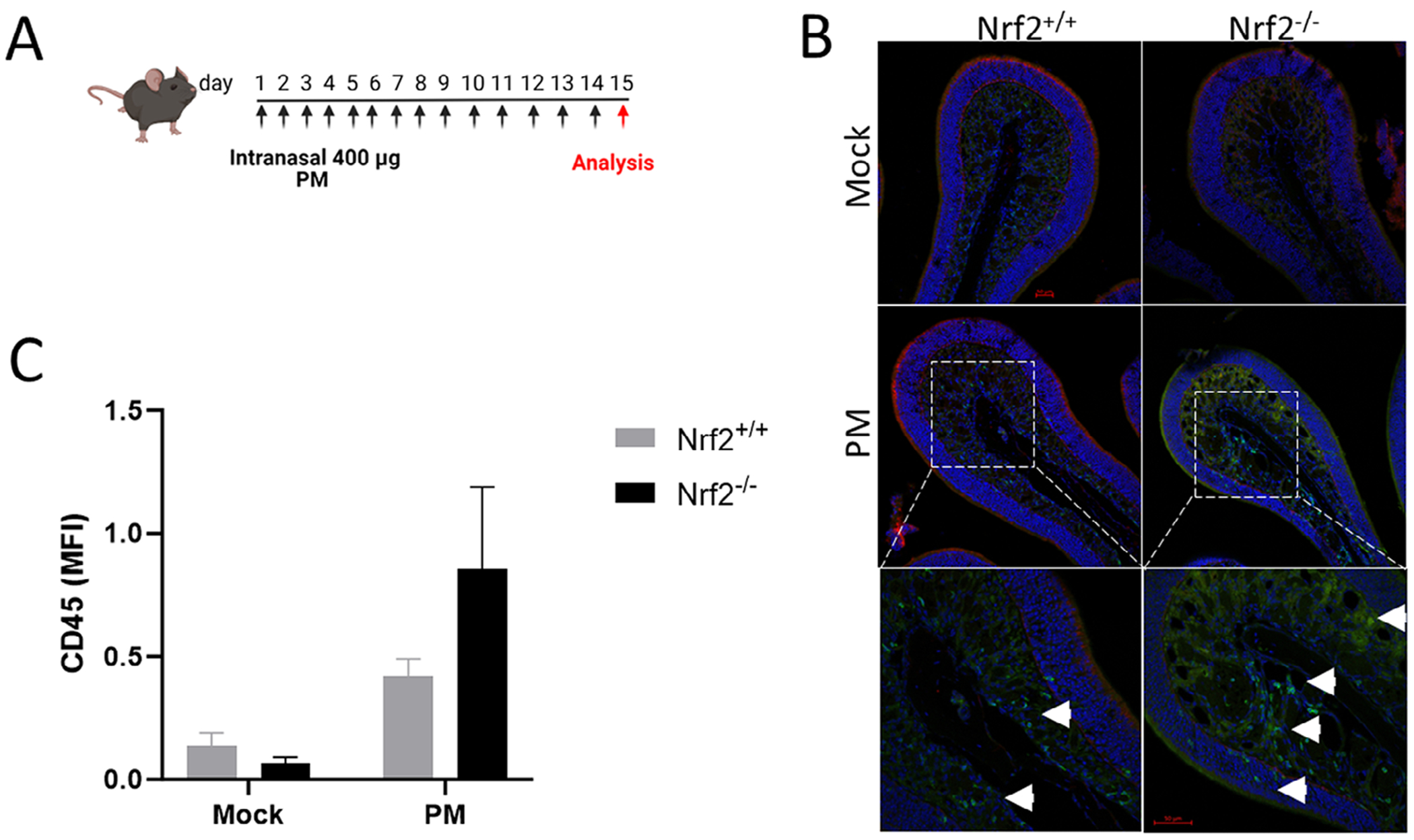
Exaggerated sinonasal inflammation in Nrf2-deficient mice. (A) Mice were exposed intranasally with PM daily for 14 days and analyzed for sinonasal inflammation by immunofluorescence. (B) Representative confocal images of the olfactory turbinate frommice on day 15. Blue: DAPI, Red: Krt5, Green: CD45. (C) Median fluorescence intensity (MFI) of CD45 in olfactory turbinates. Data are presented as mean ± SEM. *N* = 2 per group.

To assess the role of Nrf2 in regulating sinonasal inflammation following in vivo PM exposure, Nrf2^+/+^ and Nrf2^−/−^ mice were subjected to daily intranasal PM exposure for 14 days and analyzed by immunofluorescence on day 15 (Figure 1A). Following this exposure model, Nrf2^−/−^ mice exhibited a greater degree of CD45^+^ cell infiltrate in the olfactory mucosa compared to Nrf2^+/+^ mice, but did not achieve statistical significance (Figure 1B,C).

### 3.2 | Nrf2^−/−^ Mice Exhibit Increased Mucin Production Compared to Nrf2^+/+^ Mice

**FIGURE 2 |:**
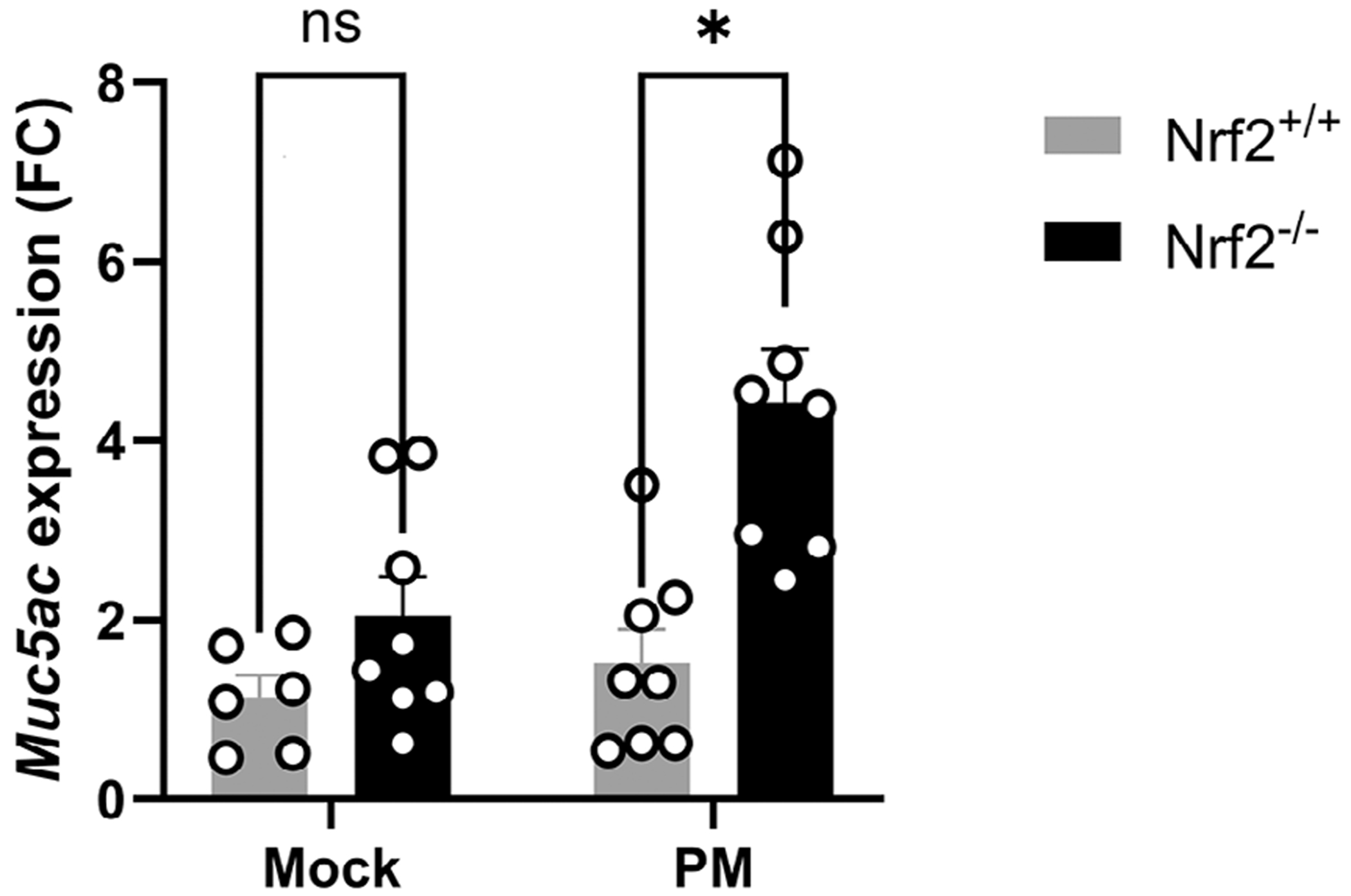
Nrf2 deletion increasesmucin synthesis in the sinonasal epithelium. (A) RNA was isolated from mice on day 15 and *Muc5ac* levels were analyzed by qPCR. Data are presented as mean ± SEM of fold change (FC) relative to baseline. *N* = 8–10 per group. *p* value calculated by Student’s *t*-test. *p < 0.05, NS: non-significant.

Mucous overproduction and goblet cell metaplasia is a hallmark of CRS. We next sought to examine the role of Nrf2 in PM-induced mucous production in vivo by measuring mRNA levels of the mucin *Muc5ac*. We found that expression of *Muc5ac* is significantly elevated in Nrf2^−/−^ compared to Nrf2^+/+^ mice (Figure 2).

### 3.3 | Nrf2 Deletion Worsens PM-Induced Neutrophilic Inflammation

**FIGURE 3 |:**
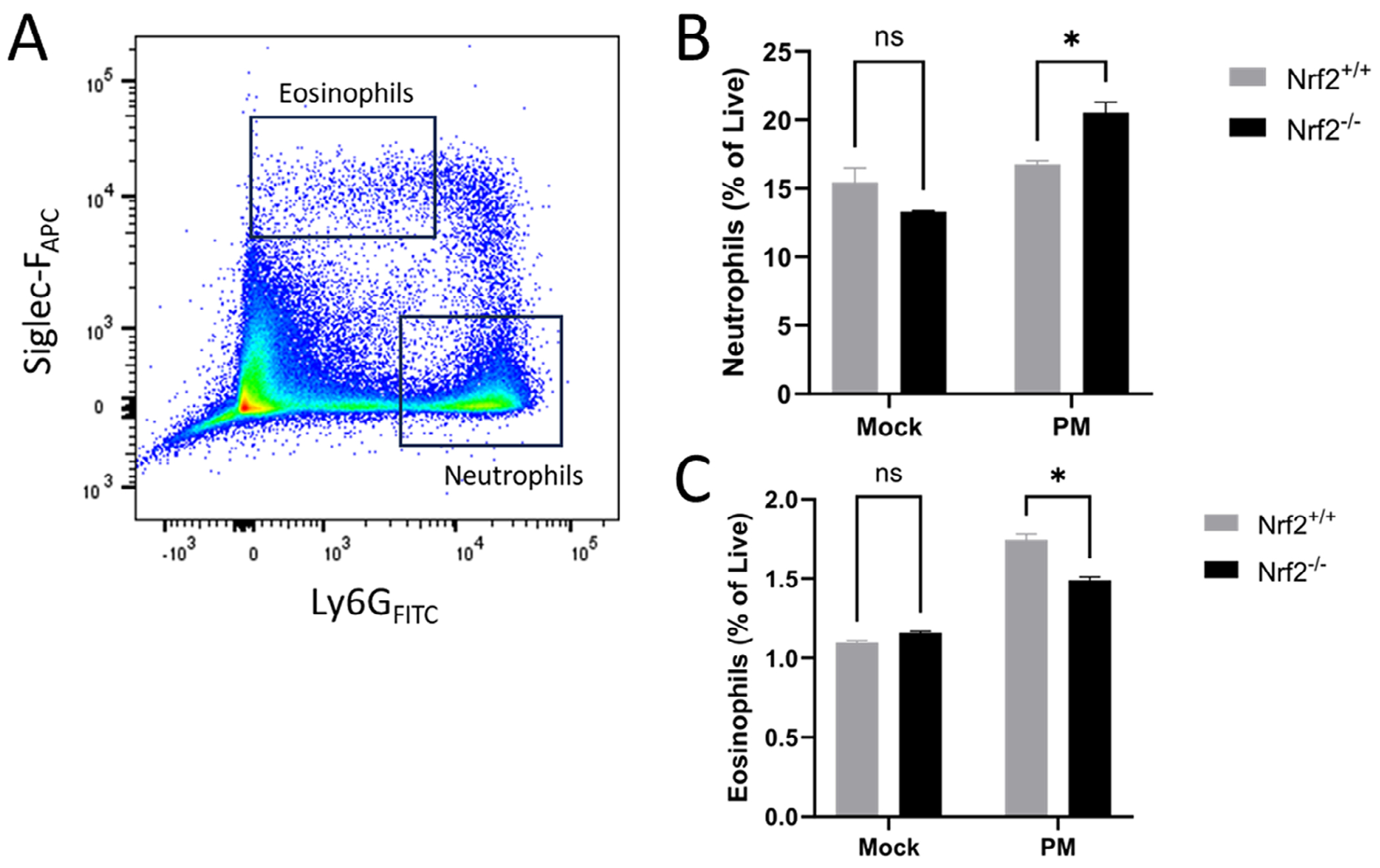
Nrf2 deficiency promotes neutrophilic infiltrate following PM exposure. (A) Day 15 mice were euthanized and nasal mucosa was harvested and analyzed by flow cytometry. Cells were analyzed for Ly6G and Siglec-F surface expression. (B) Sinonasal mucosa neutrophils (Siglec-F^−^, Ly6G^+^) and (C) eosinophils (Siglec-F^+^, Ly6G^intermediate^) were quantified as a percentage of live cells. Data are presented as mean ± SEM. *N* = 2 per group. *p < 0.05 by Student’s *t*-test.

We next examined the type of inflammatory response that Nrf2 deficiency exacerbates in PM-exposed mice. Work by our group has shown that Nrf2 deletion increases inflammation in models of allergic sinonasal inflammation [19]. Therefore, we analyzed both neutrophilic and eosinophilic infiltrate in the sinonasal mucosa of PM-exposed mice (Figure 3A). Flow cytometric analysis revealed that Nrf2 deficiency significantly exacerbates neutrophilic sinonasal infiltrate, but led to a slight decrease in eosinophilic inflammation (Figure 3B,C).

### 3.4 | Nrf2 Regulates Type 2 Cytokine Expression Following PM Exposure

**FIGURE 4 |:**
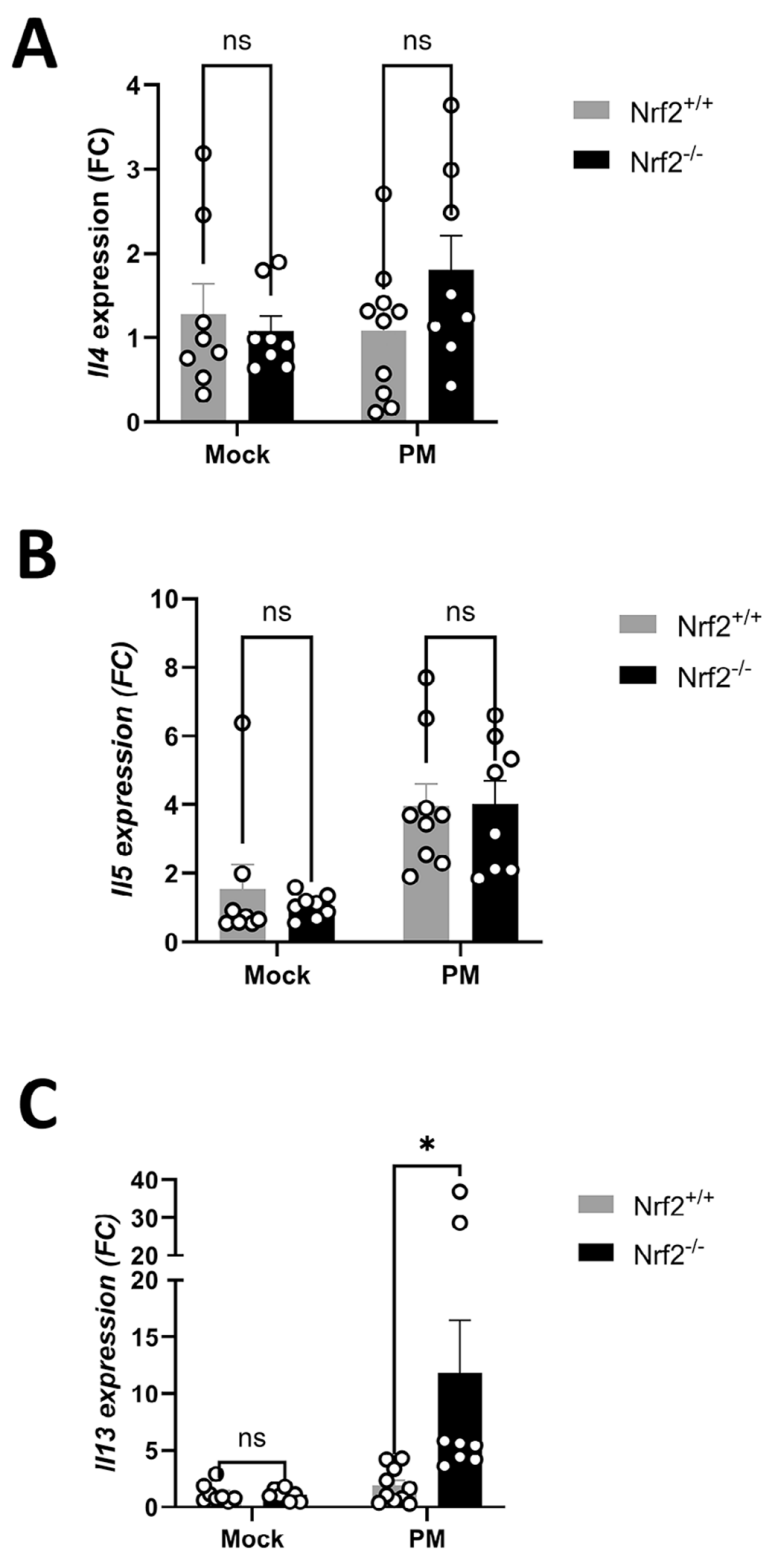
IL-13 expression is elevated in sinonasal mucosa from Nrf2 knockout mice. (A) RNA was isolated from mice on day 15 and *Il4*, (B) *Il5*, and (C) *Il13* levels were analyzed by qPCR. Data are presented as mean±SEM of fold change (FC) relative to baseline.*N*=8–10 per group. *p* value calculated by Mann–Whitney *U* test. *p < 0.05, NS: non-significant.

The observation that Nrf2^−/−^ mice exhibit increased mucin production led us to next examine the expression of type 2 cytokines, which are associated with goblet cell metaplasia [25]. qPCR analysis from sinonasal mucosal tissue from Nrf2^+/+^ and Nrf2^−/−^ mice following 2 weeks of PM exposure revealed that Nrf2-deficient mice exhibit significantly elevated expression of *Il13* but similar expression of *Il4* and *Il5* compared to Nrf2 wild-type mice (Figure 4A,C).

### 3.5 | Epithelial Alarmin Expression Is Elevated in Nrf2 Knockout Mice

**FIGURE 5 |:**
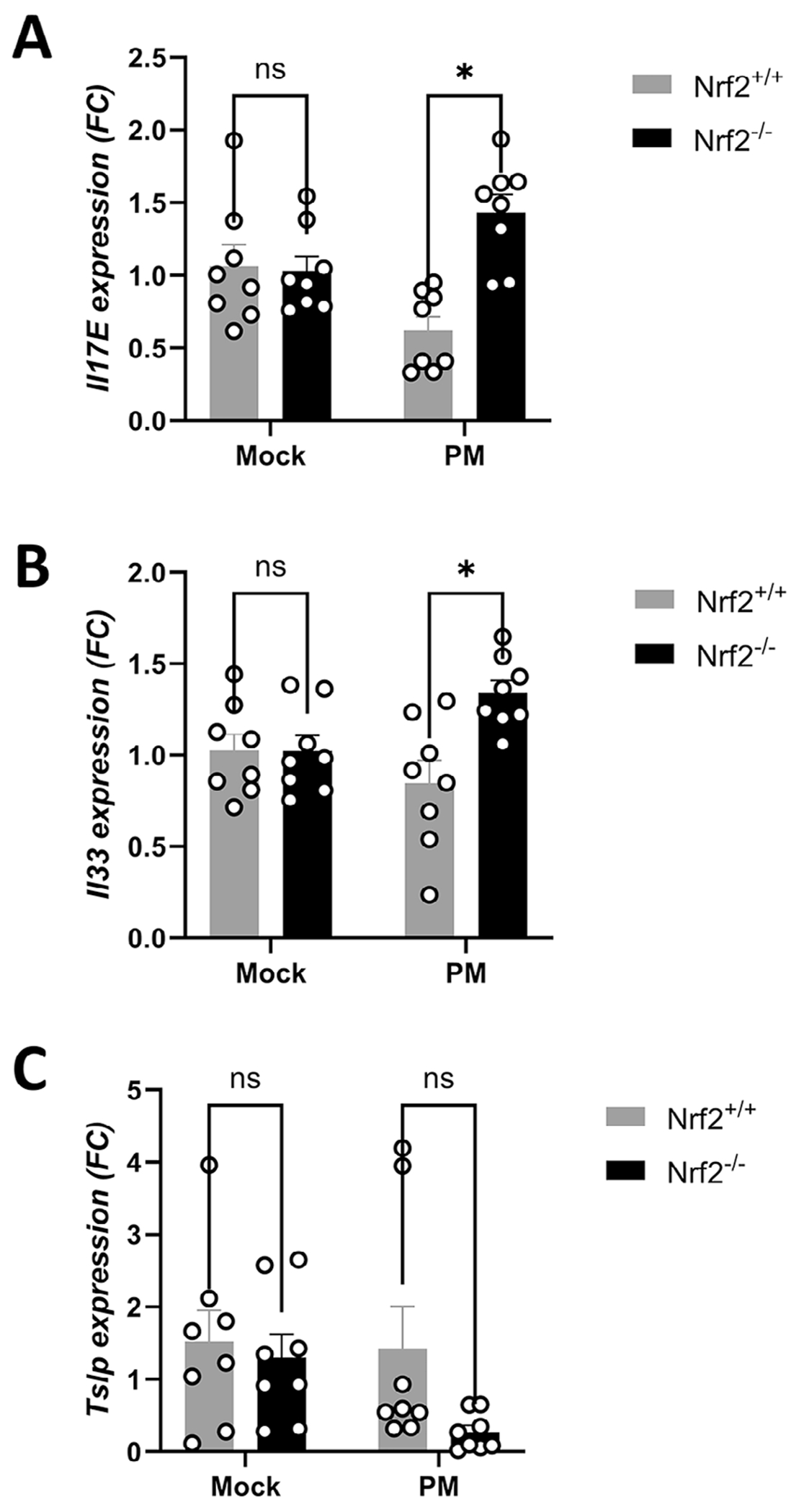
Nrf2 deletion increases transcription of epithelial-derived alarmins. (A) RNA was isolated from mice on day 15 and *Il17E*, (B) *Il33*, and (C) *Tslp* levels were analyzed by qPCR. Data are presented as mean ± SEM of fold change (FC) relative to baseline. *N* = 8–10 per group. *p* value calculated by Student’s *t*-test. *p < 0.05, NS: non-significant.

Additional drivers of sinonasal inflammation are alarmins, which are epithelial-derived cytokines which can initiate and propagate innate immune responses. We therefore examined the expression of these alarmins in Nrf2^+/+^ and Nrf2^−/−^ mice on day 15 after PM exposure. Nrf2 knockout mice showed significantly increased expression of the alarmins *ll33* and *Il17e*, which encode the protein IL-25 (Figure 5A,B). *Tslp* expression, however, was significantly decreased in Nrf2^−/−^ mice (Figure 5C).

### 3.6 | Type 3 Cytokines Are Increased in Nrf2-Deficient Mice Following PM Exposure

**FIGURE 6 |:**
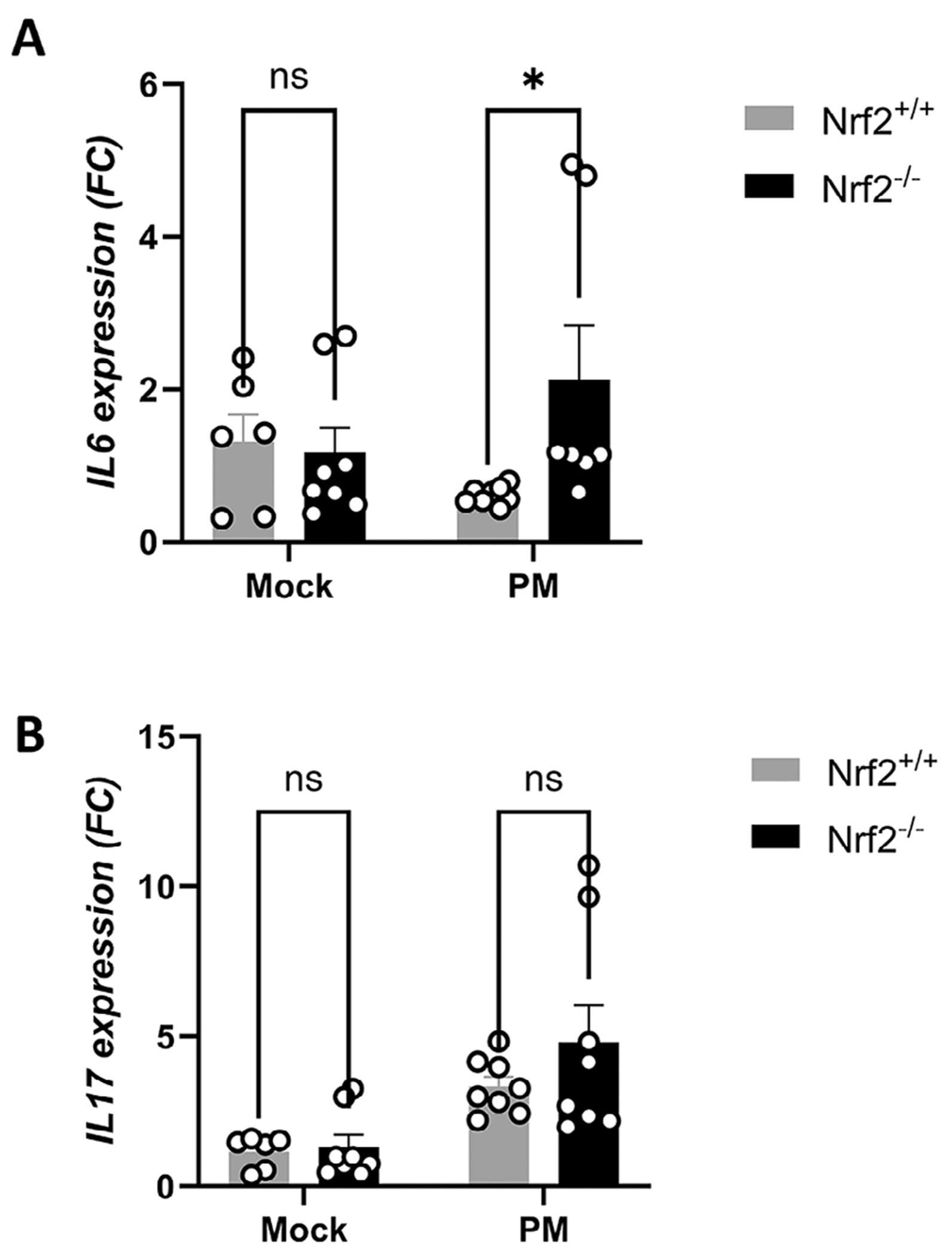
Type 3 cytokines are elevated in PM-exposed Nrf2-deficient mice. (A) RNA was isolated from mice on day 15 and *Il6*, and (B) *Il17A* levels were analyzed by qPCR. Data are presented as mean ± SEM of fold change (FC) relative to baseline. *N* = 8–10 per group. *p* value calculated by Mann–Whitney *U* test. *p < 0.05, NS: non-significant.

The finding that Nrf2^−/−^ mice exhibit elevated neutrophilic infiltrate in the sinonasal mucosa, but not increased eosinophilic infiltrate, led us to next examine mucosal type 3 cytokine expression. Type 3 cytokines are mediators involved in Th17 immune responses, which are characterized by profound neutrophilic inflammation [26]. qPCR analysis revealed that PM exposure in Nrf2^−/−^ mice induced significantly greater levels of *Il6* and a trend toward increased *Il17A* than in Nrf2^+/+^ mice (Figure 6A,B).

## 4 | Discussion

CRS is a highly prevalent disorder with heterogeneous pathophysiology and unclear etiology. Environmental exposures, such as airborne PM, have been closely linked to CRS prevalence and severity [6, 27, 28]. Animal models of allergic sinonasal inflammation have demonstrated that PM exposure exacerbates existing type 2 inflammation, suggesting an adjuvant property of PM_2.5_ in allergic disease [29–31]. More recent studies have demonstrated that PM exposures, in the absence of allergens or other bystander antigens, in animals yield a phenotype similar to CRSsNP, characterized by neutrophilic inflammation and type 1 and type 3 immune mechanisms [11, 12].

PM consists of heavy metals and polycyclic hydrocarbons, which promote cellular oxidative stress [7, 14]. This oxidative stress disrupts the epithelial barrier in the upper respiratory tract, allowing for the passage of chemical irritants leading to inflammation [19, 21]. Our group has previously demonstrated that activation of the Nrf2 pathway, a key regulator of the endogenous cellular antioxidant response, prevents this PM-induced barrier dysfunction [21]. Thus, we hypothesized that genetic deletion of Nrf2 in a murine model of subacute airway PM exposure would exacerbate sinonasal inflammation.

In this study, we demonstrate that mice harboring an Nrf2 deletion with short-term exposure to PM exhibited significantly increased inflammatory cellular infiltrate in the sinonasal mucosa, which consisted predominantly of neutrophils. Interestingly, Nrf2 deletion led to a slight decrease in tissue eosinophilia, suggesting that the effect of Nrf2 on adaptive immune responses may be context dependent. This decrease in eosinophils, however, represented a small (<5%) of the overall cellular infiltrate. This difference may be attributable to differences in eotaxin expression or intrinsic changes in Nrf2 deficient eosinophils, which may represent a promising avenue for research in the context of type 2 inflammatory disorders. Further, Nrf2 deletion increased expression of mucin genes in the upper respiratory tracts measured by *Muc5ac* transcription, which correlates with goblet cell metaplasia [32]. However, protein levels of mucins were not directly measured in our analysis. Finally, we show that deletion of Nrf2 increases synthesis of epithelial-derived cytokines, IL-13, IL-6, and IL-17A. Loss of Nrf2, somewhat surprisingly, led to a decrease in TSLP expression. Much of the published literature demonstrates that TSLP is a potent inducer of type 2 immune responses in allergic disease [33]. Recent studies investigating the role of TSLP in Th17-mediated gastrointestinal disease, however, suggest that TSLP may play a role in suppressing Th17 cell differentiation [34].

Collectively, these data demonstrate that Nrf2 is a key regulator of PM-induced type 3 sinonasal inflammation. Nrf2, therefore, represents a promising therapeutic target for the treatment of CRSsNP. At present, first-line therapy for CRSsNP consists of topical steroids applied via topical spray, nasal irrigation, sinonasal catheter, or exhalation delivery systems [35, 36]. A recent meta-analysis demonstrated that CRSsNP patients without allergy have mixed responses to topical steroid therapy, with no improvement following steroid nasal spray modalities and only a modest response to exhalation delivery systems [37]. This indicates a significant need for new, highly effective therapies for CRSsNP.

Our study utilizes a genetic deletion of Nrf2 and demonstrates that Nrf2 plays a biologically significant role in regulating PM-induced type 3 sinonasal inflammation. To further establish the therapeutic potential of the Nrf2 pathway in CRS, future studies may use genetic ablation of Keap1, the constitutive inhibitor of Nrf2 activity, or Nrf2 activating chemical compounds [38]. Several Nrf2 activating compounds have been validated, including naturally occurring compounds like sulforaphane or synthetic molecules like triterpenoids and fumaric acid esters [20, 39]. Administering these compounds in the PM exposure model described here may provide further validation of Nrf2 as a potential therapeutic target for CRS.

In conclusion, this is the first study to our knowledge to demonstrate that Nrf2 regulates short-term PM-induced sinonasal inflammation. Although previous studies have examined long-term exposures, the findings in this study also highlight that even short-term PM exposure may create an inflammatory response in the nose and sinuses. These findings warrant further studies to better understand the molecular mechanisms of how short-term PM exposure induces sinonasal inflammation.
